# CircZFR serves as a prognostic marker to promote bladder cancer progression by regulating miR-377/ZEB2 signaling

**DOI:** 10.1042/BSR20192779

**Published:** 2019-12-04

**Authors:** Wen-Yuan Zhang, Qing-Hong Liu, Tie-Jun Wang, Jun Zhao, Xiao-Hua Cheng, Jin-Shan Wang

**Affiliations:** 1 Department of Urology, East Hospital, Ji’an Hospital, Jiangxi 343000, China; 2 Department of Urology, East Hospital, Tongji University School of Medicine, Shanghai 200123, China

**Keywords:** Bladder cancer, CircZFR, miR-377, Prognostic biomarker, ZEB2

## Abstract

Circular RNAs (circRNAs) have been identified as crucial regulators of gene expression in human cancer biology. CircZFR is a novel identified circRNA and its effect in bladder cancer remains unclearly. In the present study, we aimed to investigate the role of circZFR in the progression of bladder cancer. First, we demonstrated that the expression of circZFR was higher in bladder cancer tissues and cells compared with adjacent non-tumor tissues and normal bladder epithelial cells. And higher circZFR levels were positively correlated with bladder cancer patients’ pathological T stage, grade, lymphatic metastasis, recurrence, progression-free survival (PFS) and overall survival (OS). Functionally, knockdown of circZFR could significantly prohibit cell growth, migration and invasion, arrest cell cycle as well as promote apoptosis of bladder cancer cells *in vitro* study. Mechanistically, we observed that circZFR could directly bind to miR-377 as sponge to promote ZEB2 expression in bladder cancer cells. In addition, rescue assays demonstrated that restoration of ZEB2 significantly impaired the suppressive effects of circZFR silencing on bladder cancer cells growth, migration and invasion. Taken together, our results illuminated that circZFR could be a prognostic biomarker in bladder cancer and exerted oncogenic roles through regulating miR-377/ZEB2 axis in bladder cancer, which indicated that circZFR could be a potential therapeutic target for bladder cancer patients treatment.

## Introduction

Bladder cancer (BC) is the most prevalent malignancy of the urinary system worldwide, with the highest morbidity and mortality among urinary system tumors in China [1]. Bladder cancer consists of 75% non-muscle invasive bladder cancer (NMIBC) and 25% muscle-invasive bladder cancer (MIBC) according to the depth of tumor infiltration [2]. MIBC patients usually present with local or distant metastasis and poor prognosis. Although radical cystectomy, systemic chemotherapy, target-therapy and immunotherapy have greatly advanced the treatment of BC, the overall survival rate remains unsatisfactory due to the high recurrence and distant metastasis [3]. Therefore, identifying novel biomarkers and potential therapeutic targets underlying the tumorigenesis and progression of BC is imperative for building up therapy strategies for BC.

Circular RNAs (circRNAs), a new member of non-coding RNAs, are characterized with a covalently closed continuous loop structure [4]. And more and more circRNAs have been identified as endogenous non-coding RNAs through high-throughput sequencing technology in various cell lines and species. Importantly, recent reports have proved that circRNAs are involved in the initial, development and progression of many disease including cancer [5,6]. Further, a large amount of studies demonstrate that circRNAs exhibit crucial roles, and may be potential biomarkers and therapeutic targets in cancers [7].

CircZFR, a new circRNA (circBase ID: hsa_circ_0072088, hsa_circRNA_103809) mapping to chromosome 5p13.3, functions as tumor suppressor or oncogene in different cellular context. For instance, Tan et al. found that circZFR promotes hepatocellular carcinoma progression through regulating miR-3619-5p/CTNNB1 axis and activating Wnt/β-catenin pathway [8]. CircZFR contributes to cancer cell proliferation and invasion in papillary thyroid cancer and lung cancer by sponging miRNA to regulate the target gene expression [9,10]. Conversely, circZFR inhibits the cell proliferation and migration in colorectal cancer via miR-532-3p/FOXO4 axis [11]. Further, Liu et al. showed that circZFR prohibits cell proliferation and promotes apoptosis in gastric cancer by sponging miR-130a/miR-107 and modulating PTEN [12].

However, the function and mechanism of circZFR in the development and progression of BC has not been explored deeply. In the present study, we characterized the expression of circZFR in BC tissues and explored its clinical significance and biological effects in BC. To the best of our knowledge, these data demonstrated for the first time the oncogenic role of circZFR in BC, indicating circZFR might be a potential prognostic biomarker and therapeutic target in BC.

## Materials and methods

### Patients and sample collection

A total of 104 pairs of BC tissues and adjacent normal tissues were collected from BC patients who received treatment in Shanghai East Hospital, Tongji University School of Medicine between January 2013 and January 2016. None of the patients have received any preoperative local or systemic treatment before specimen collection. Written informed consent was acquired from each patient or their relatives and the project was approved by the Board and Ethics Committee of Shanghai East Hospital, Tongji University School of Medicine. The collected tissues were directly deposited in liquid nitrogen until further use.

### Cell lines and culture conditions

The normal bladder epithelial cells CCC-HB-2 and seven BC cell lines (UMUC3, T24, J82, 5637, SW780, EJ and BIU87) were purchased from American type culture collection (ATCC) (Maryland, U.S.A.). All cell lines were cultured in RPIM-1640 medium (GIBCO, Thermo Fisher Scientific, Inc., Waltham, MA, U.S.A.) supplemented with 10% fetal bovine serum 1% penicillin/streptomycin (HyClone; GE Healthcare Life Sciences, Logan, UT, U.S.A.). All cell lines were cultured in humidified incubator at 37°C with 5% CO_2_.

### RNA preparation and qRT-PCR

The total RNA from the tissues and cells was extracted with the Trizol reagent (Thermo Scientific, U.S.A.), according to the manufacturer’s guide. The quantity of RNA was detectedby Nanodrop spectrophotometer (Thermo Scientific, U.S.A.). Reverse transcription was done with a Reverse Transcription Kit (TaKaRa, Japan) for circRNA and mRNA according to the protocol. Subsequently, quantitative real-time PCR (qRT-PCR) was performed with the Mx3005P QPCR Systems (Agilent Technologies, Inc., U.S.A.), according to the manufacturer’s instructions. GAPDH and small nuclear U6B were used as an endogenous control for circRNA and miRNA, respectively. The primers were constructed by Biosune (Shanghai, China). The relative quantity of target gene was calculated with the 2^−ΔΔ*C*t^ methods.

### Cell proliferation assay

For the cell viability assay, cells transfected with siRNA were plated into 96-well tissue culture plates (3 × 10^3^ cells per well), and cell viability was evaluated with MTS (Promega, U.S.A.), according to the manufacturer’s protocol. The absorbance value was measured at a wave length of 492 nm using MicroplateReader (Multiskan Sky, Thermo Fisher Scientific). Experiments were performed in triplicate. For cell colony formation detection, soft agar assay was performed using the CytoSelect™ 96-Well Cell Transformation Assay (Standard Soft Agar kit, Cell Biolabs), according to the manufacture’s instruction. Cells transfected with siRNA were seeded into a 96-well plate (5 × 10^3^ cells per well). After incubation in a semisolid agar medium at 37°C in a 5% humidified atmosphere for 2 weeks, colonies are then hydrolyzed and measured with the CyQuant, and the relative absorbance value was detected with Envision (PerkinElmer). Three independent experiments were performed in triplicate.

### Migration and invasion assay

Cell migration or invasion was measured with BD 24-well transwell chamber (Costar, Massachusetts, U.S.A.) with or without pre-coated Matrigel according to manufacturer’s guide. Cells (6 × 10^4^ cells per well for migration; 8 × 10^4^ cells per well for invasion) in 500 μl of serum-free medium were seeded to the insert, and media supplemented with 10% FBS as chemoattractant were placed in the lower chamber of transwell. After incubating 24 h at 37°C, the invaded cells at lower surface of the insert were fixed, stained with 1% Crystal Violet, calculated and photographed.

### Flow cytometry assay

Cell apoptosis and cell cycle were determined by flow cytometry assay as previous study [13]. For apoptosis detection, the cells were stained with Annexin V-FITC Apoptosis Kit (BD Biosciences), according to the manufacturer’s protocal. Briefly, cells were digested, then washed with cold PBS, and diluted in 100 μl binding buffer. Fluorescein isothiocyanate (FITC) and propidium iodide (PI) stained the cell for 15 min at indoor temperature away from light. Finally, the results were determined by flow cytometry (FACS Calibur, BD Biosciences). For cell cycle analysis, cells transfected with siRNA were obtained for 48 h and subsequently fixed with 70% ethanol at –20°C overnight, dyed using PI added with Ribonuclease A (Sigma) for 30 min at indoor temperature. Flow cytometry was used to analyze the cell cycle distribution (FACS Calibur, BD Biosciences).

### Biotin-labeled pull-down assay

Briefly, 1 × 10^7^ BC cells were harvested, lysed in RIP lysis buffer, and incubated with the biotinylated circZFR probe-coated M280 streptavidin dynabeads (Invitrogen) at 4°C overnight. Subsequently, the RNA complexes bound to the beads were purified using Trizol Reagent (Invitrogen) for further qRT-PCR analysis.

### Luciferase reporter assay

For luciferase reporter assay, circZFR-WT, circZFR-mutant, ZEB2-WT and ZEB2-mutant were constructed into pGL3-Basic luciferase vector (GenePharma, Shanghai, China) to construct reporter plasmids. Afterwards, cells were co-transfected with indicated reporter plasmids and microRNA using Lipofectamine 2000 (Life Technologies). Subsequently, the relative luciferase activity was detected by the dual-luciferase reporter assay system (Promega, Madison, WI, U.S.A.) at 48 h post-transfection. Firefly luciferase activity was normalized to renilla luciferase activity.

### Western blot analysis

RIPA buffer was used to lyse the cells. BCA protein assay (Thermo Fisher Scientific, U.S.A.) was used to detect the protein level. SDS-PAGE was performed to separate the protein, and subsequently the protein was transferred to PVDF membranes, and incubated with antibodies overnight. Finally, the intensity of band was detected by enhanced chemiluminescence reagent (32109; Thermo Fisher Scientific). The antibodies included anti-GAPDH antibody (1:1000 dilution; Sigma); anti-ZEB2 antibody (ab138222, Abcam).

### Statistical analysis

Statistical analysis was performed by GraphPad Prism 7.0(GraphPad, La Jolla, CA, U.S.A.), and data were expressed as mean ± SD (standard deviation, SD). The chi-square tests were applied to explore the correlations between circZFR expression and clinicopathological factors. Student’s *t*-test was used to determine significant difference between two groups. Kaplan–Meier method and the log-rank test was used to analyze the PFS and OS between two groups. Univariate and multivariate Cox proportional hazards model was performed to identify factors that were significantly related with PFS and OS. Receiver operating characteristic (ROC) curve analysis was plotted to assess the predictive value of the circZFR for the diagnosis of this disease. *P* < 0.05 was considered as significant criteria.

## Results

### CircZFR was remarkably up-regulated in BC tissues and cell lines and related with poor prognosis of BC patients

**Figure 1:**
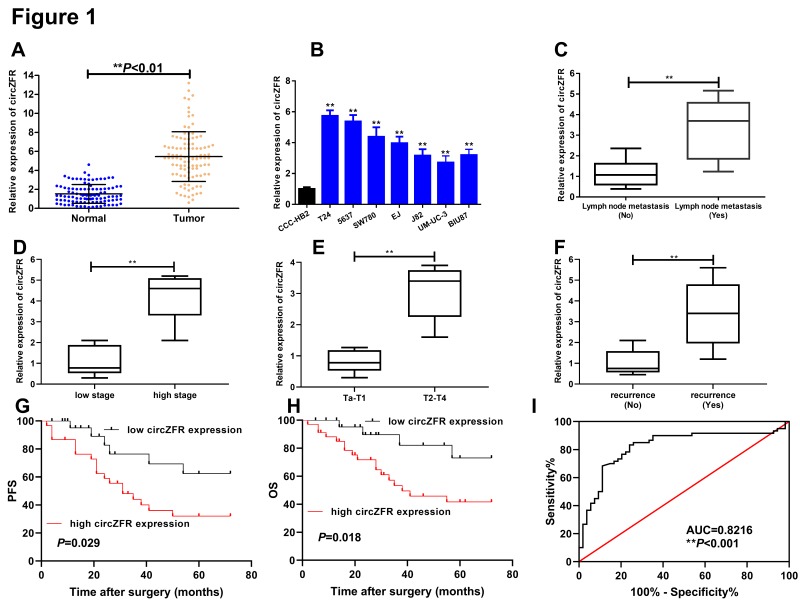
Up-regulated circZFR in bladder cancer and its association with poor prognosis of bladder cancer patients (**A**) The high expression levels of circZFR in 104 paired bladder cancer tissues compared with adjacent normal tissues by qRT-PCR (**P < 0.01). (**B**) The expression of circZFR in bladder cancer cell lines and human normal bladder epithelial cell (CCC-HB-2), **P < 0.01 compared with CCC-HB-2. (**C–F**) The expression levels of circZFR in bladder cancer patients with lymph node status, grades, pathological T stages, recurrence status. (**G** and **H**) Kaplan–Meier analysis and log rank test showed that patients with high circZFR expression had short progression-free survival (PFS) and overall survival (OS). (**I**) The receiver operating characteristic (ROC) curve showed the diagnostic value by using circZFR expression.

To investigate the role of circZFR in BC, we detected the expression of circZFR in BC tissues and the adjacent normal tissues from 104 BC patients as well as BC cells by qRT-PCR. As shown in Figure 1A, circZFR expression was strongly increased in BC tissues compared with adjacent normal tissues (Figure 1A). Consistently, circZFR expression was also significantly up-regulated in seven BC cell lines (UMUC3, T24, J82, 5637, SW780, EJ and BIU87) compared with normal bladder epithelial cell (CCC-HB-2) (Figure 1B). To better understand prognostic role of circZFR, we observed that high expression of circZFR was obviously correlated with and lymph node metastasis (Figure 1C) high stage (Figure 1D), highly pathological T stage (Figure 1E), recurrence (Figure 1F). Also, the correlation analysis demonstrated that circZFR expression was associated with clinicopathological features including the tumor stage, grade, lymphatic metastasis, recurrence (Table 1) (the expression of circZFR ≥ the median was defined as high expression, the expression of circZFR < the median was defined as low expression according to published study [14]). In addition, Kaplan–Meier analysis reported that BC patients with high circZFR expression showed poorer progression-free survival (PFS) (Figure 1G, *P* = 0.029) and overall survival (OS) (Figure 1H) compared with the patients with low circZFR expression. Furthermore, ROC curve analysis indicated that circZFR acted as diagnostic biomarker of BC patients (Figure 1I, AUC = 0.8216).

**Table 1:** Relationship between the expression levels of circZFR and clinicopathological features in bladder cancer

Characteristics	No.	circZFR expression		
		High	Low	*P*-value
Gender				
Male	78	40	38	0.651
Female	26	12	14	
Age				
<60	38	16	22	0.222
≥60	66	36	30	
Tumor size				
<3 cm	60	32	28	0.427
≥3 cm	44	20	24	
Tumor stage				
Ta-T1	40	6	34	<0.001***
T2-T4	64	46	18	
Grade				
Low	34	8	30	<0.001***
High	70	44	22	
Lymphatic metastasis				
Positvie	55	35	20	0.003**
Negative	49	17	32	
Vascular invasion				
Yes	19	9	10	0.780
No	85	43	42	
Recurrence				
Yes	44	32	12	<0.001***
No	60	20	40	
Total	104	52	52	

Chi-square test. **P < 0.01, ***P < 0.001.

### Silencing of circZFR inhibited cell growth, migration and invasion of BC cells *in vitro*

**Figure 2:**
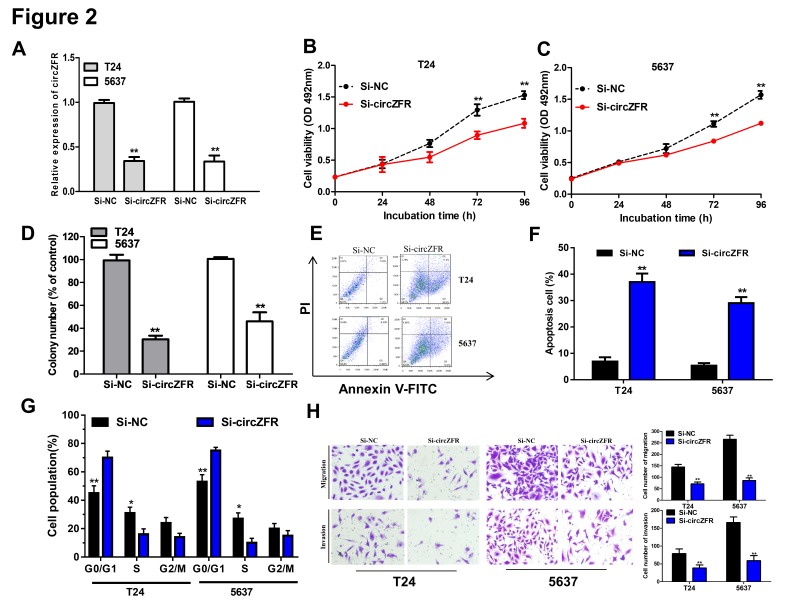
circZFR exerted oncogenic effect in bladder cancer cells (**A**) Expression of circZFR was confirmed by qRT-PCR in T24 and 5637 cells transfected with Si-NC or Si-circZFR. **P < 0.01 compared with Si-NC. (**B** and **C**) Konckdown of circZFR significantly inhibited cell proliferation of T24 and 5637 cells by MTS assay. **P < 0.01 compared with Si-NC. (**D**) Soft-agar assay was used to detect the colony-forming ability of T24 and 5637 cells transfected with Si-NC or Si-circZFR. **P < 0.01 compared with Si-NC. (**E** and **F**) Cell apoptosis was determined by flow cytometry in T24 and 5637 cells transfected with Si-NC or Si-circZFR. **P < 0.01 compared with Si-NC. (**G**) Cell-cycle was determined by flow cytometry in T24 and 5637 cells transfected with Si-NC or Si-circZFR. (**H**) Transwell assay was used to detect the migration and invasion of T24 and 5637 cells transfected with Si-NC or Si-circZFR. **P < 0.01 compared with Si-NC. Data were expressed as mean ± SD from three independent assay. *P < 0.05 compared to Si-NC; **P < 0.01 compared with Si-NC.

To investigate whether circZFR could influence the biological function of BC cells, we selected T24 and 5637 cells (the highest expression of circZFR among BC cell lines, Figure 1B) to perform the loss-of-function assays. First, we confirmed that circZFR was successfully knockdown after siRNA transfection by qRT-PCR assay analysis (Figure 2A). Subsequently, MTS assay and soft agar colony formation assay revealed that knockdown of circZFR significantly suppressed cell growth and colony-forming abilities in T24 and 5637 cells (Figure 2B–D). To further detect whether circZFR could affect the apoptosis and cell cycle of BC cells, we performed the flow cytometry assay and the results revealed that circZFR knockdown prominently promoted cell apoptosis (Figure 2E,F) and arrested cell cycle at G0/G1 stage (Figure 2G) in T24 and 5637 cells. Next, transwell assay revealed that circZFR knockdown could significantly attenuate the migration and invasion abilities of BC cells (Figure 2H).

### circZFR sponged miR-133a and promoted ZEB2 expression

**Figure 3:**
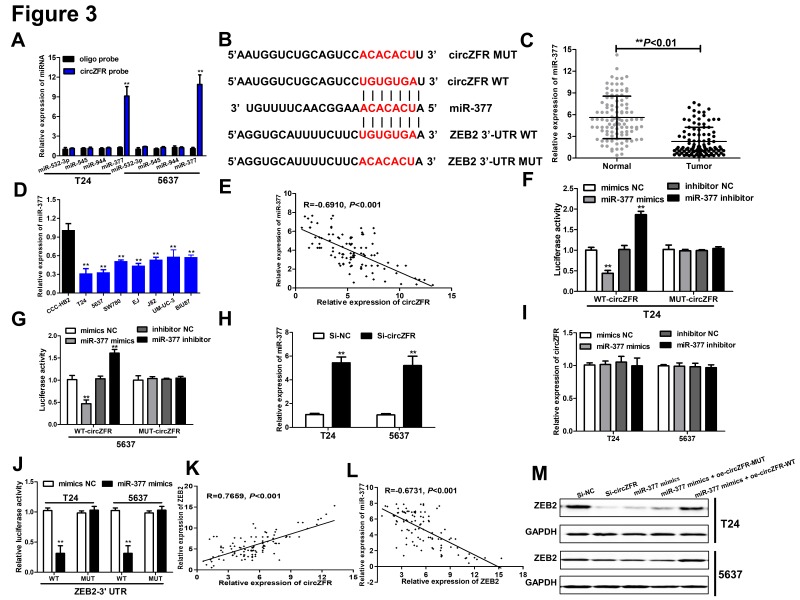
CircZFR directly interacted with miR-377 and promoted ZEB2 expression (**A**) qRT-PCR was used to determine the expression of miRNAs in the bladder cancer cells pulled down by biotinylated circZFR probe. (**B**) Predicted binding sites of miR-377 in circZFR and ZEB2 3’-UTR. (**C**) The relative expression of miR-377 in tumor tissues was significantly down-regulated compared with that in adjacent normal tissues. (**E**) Negative correlation between miR-377 and circZFR expression in bladder cancer tissues by Pearson’s correlation analysis. (**D**) The expression of miR-377 in bladder cancer cells was significantly dereased by qRT-PCR. (**F** and **G**) The relative luciferase activities were prohibited in the T24 and 5637 cells transfected with the reporter vector WT-circZFR, but not MUT-circZFR. (**H**) The expession of miR-377 was down-regulated by qRT-PCR in T24 and 5637 cells transfected with Si-circZFR. (**I**) The expession of circZFR was determined by qRT-PCR in T24 and 5637 cells transfected with NC, miR-377 inhibitor, miR-377 mimics. (**J**) The relative luciferase activities were prohibited in the T24 and 5637 cells transfected with the reporter vector WT-ZEB2, but not MUT-ZEB2. (**K**) Correlation analysis between circZFR and ZEB2 expression. (**L**) Correlation analysis between ZEB2 and miR-377 expression. (**M**) Both circZFR koncdown and miR-377 overexpression abrogated the protein levels of ZEB2 in T24 and 5637 cells, whereas circZFR-WT overexpression inhibited the effects of miR-377. Data were expressed as mean ± SD from three independent experiments; *P < 0.05, **P < 0.01.

It had been reported that circRNAs modulated miRNA expression by sponging miRNA. To address whether circZFR sponged miRNA to regulate the progression of BC cells, potential miRNAs associated with circZFR were predicted by using three publicly available bioinformatics tools miRanda (http://www.micro-rna.org/microrna/home.do) Starbase (http://starbase.sysu.edu.cn/panCancer.php) and CircInteractome (https://circinteractome.nia.nih.gov/). We found out that 4 candidate miRNAs (miR-532-3p, miR-545, miR-944, miR-377), which were collectively predicted by three prediction tools and previously reported as tumor-suppressive miRNAs. Subsequently, to validate whether circZFR could serve as a binding platform for the above candidate miRNAs, the biotin-labeled pulldown assay was performed in T24 and 5637 cells. We observed a robust enrichment of miR-377 in the circZFR-probe pulled down pellet by qRT-PCR, but the amount of miR-532-3p, miR-545 and miR-944 in the circZFR-probe pulled down pellet had no obvious changes (Figure 3A). As shown in Figure 3B, the binding site and complementary sequence between circZFR and miR-377 were exhibted (Figure 3B), and it had been reported that miR-377 acted as a tumor suppressor and was low-expressed in multiple of cancer. Then we confirmed that miR-377 expression was dramatically decreased in the BC tissues (Figure 3C) and BC cells (Figure 3D). Interestingly, we observed a negative relationship between circZFR and miR-377 expression in BC tissues by Pearson’s correlation analysis (Figure 3E). Subsequently, we performed dual-luciferase reporter assay to further validate whether circZFR directly interacted with miR-377. The data revealed that miR-377 mimics could significantly abrogate the luciferase activity of the WT-circZFR rather than that of MUT-circZFR, while the miR-377 inhibitor showed the opposite results in T24 and 5637 cells (Figure 3F,G). Meanwhile, silencing of circZFR significantly enhanced the miR-377 expression (Figure 3H), while the miR-377 mimics and inhibitor didn’t induce the alteration of circZFR expression (Figure 3I), implying that miR-377 was regulated by circZFR and located at the downstream of circZFR.

In addition, the target of miR-377 was predicted by TargetScan and miRBase, and the online data showed that miR-377 harbored the matched binding site with ZEB2 (Figure 3B). To further validate the interaction between miR-377 and ZEB2, we performed luciferase reporter assay and the results confirmed that miR-377 mimics could strikingly diminish WT-ZEB2 3’-UTR activity but not MUT-ZEB2 3’-UTR activity (Figure 3J). Then, we further detected ZEB2 protein expression in bladder cancer tissues by immunohistochemistry (IHC) (Supplementary Figure S1A and B). Additionally, the positive relationship was observed between circZFR and ZEB2 expression and the negative relationship was also observed between ZEB2 and miR-377 by Pearson’s correlation analysis after the measurement of ZEB2 by qRT-PCR (Figure 3K,L). Finally, miR-377 mimics and circZFR knockdown could prominently inhibited the ZEB2 protein expression in BC cells while circZFR overexpression could counteract the inhibitory effect of miR-377 on ZEB2 expression by qRT-PCR and Western blot (Figure 3M). All these data showed that circZFR could promote ZEB2 expression by sponging miR-377 in BC cells.

### Restoration of ZEB2 impaired the inhibitory effects of circZFR knockdown on BC cell

**Figure 4:**
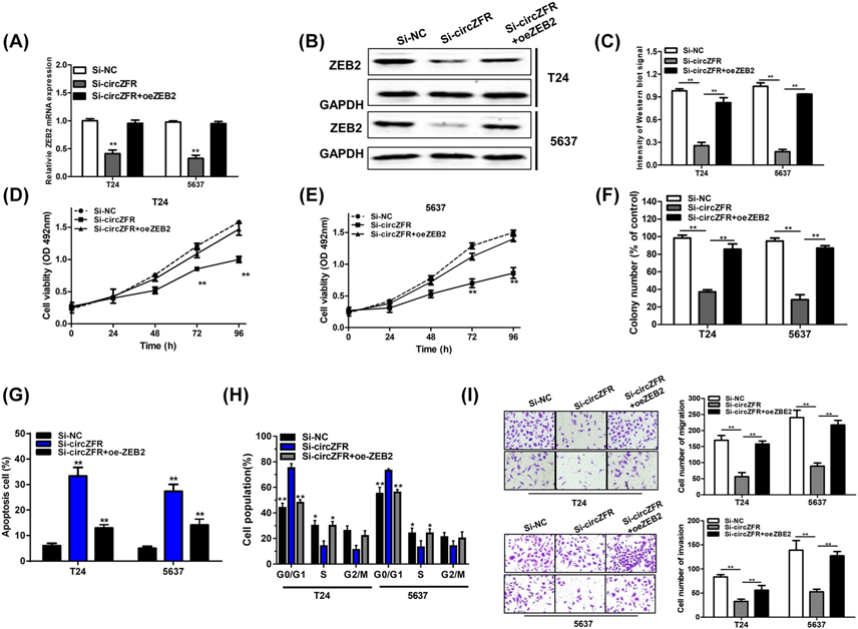
Restoration of ZEB2 impaired the inhibitory effects of circZFR silencing (**A** and **B**) The mRNA and protein expression of ZEB2 was determined in T24 and 5637 cells of circZFR knockdown. (**C**) Quantify the signal intensity of Western blots. (**D** and **E**) MTS assays demonstrated that ZEB2 overexpression could largely reverse the suppressive effects of circZFR silencing on proliferation of T24 and 5637 cells. (**F**) ZEB2 overexpression partly rescued the colony formation of circZFR silencing in T24 and 5637 cells. (**G**) ZEB2 overexpression partly abrogated the promotion of apoptosis in T24 and 5637 cells of circZFR knockdown. (**H**) ZEB2 overexpression partly rescued the cell cycle arresting in T24 and 5637 cells of circZFR silencing. (**I**) ZEB2 overexpression partly reversed the suppressive effects of circZFR silencing on migration and invasion of T24 and 5637 cells. Data were expressed as mean ± SD from three independent experiments; *P < 0.05, **P < 0.01.

As illustrated in Figure 3K,L, overexpression of circZFR significantly suppressed the inhibitory effects of miR-377 overexpression on ZEB2 expression, suggesting that circZFR might regulate the progression of BC by circZFR/miR-377/ZEB2 axis. To further confirm whether circZFR could function through regulating ZEB2 expression in BC cells, we rescued ZEB2 in the BC cells of circZFR knockdown. As shown in Figure 4A–C, ZEB2 expression was significantly restored in the BC cells of circZFR silencing. Subsequently, MTS assay and soft-agar colony formation assay revealed that ZEB2 restoration could markedly rescue the inhibitory effect of circZFR knockdown on BC growth *in vitro* (Figure 4D–F). Furthermore, ZEB2 overexpression could abrogate the promotion of apoptotic cells and the block of cell cycle in circZFR silencing BC cells (Figure 4G,H). Besides, transwell assay also demonstrated that ZEB2 overexpression could disturb inhibitory effect of circZFR knockdown on BC cell migration and invasion (Figure 4I). In conclusion, these results demonstrated that circZFR augmented BC cell growth, migration and invasion by boosting ZEB2 expression via sponging miR-377.

## Discussion

In recent years, increasing evidence suggest that circRNAs are aberrantly expressed and play a crucial role in many cancer, including lung cancer, colorectal cancer, gastric cancer, liver cancer, breast cancer and BC [5]. And an increasing number of circRNAs have been identified as one oncogene or tumor suppressor in tumorigenesis of BC [15]. For example, circular RNA circSLC8A1 acts as a sponge of miR-130b/miR-494 in suppressing BC progression via regulating PTEN [16]. Circular RNA hsa_circ_0068871 regulates FGFR3 expression and activates STAT3 by targeting miR-181a-5p to promote BC progression [17]. However, the relationship between circRNA and the initiation and progression of BC still needs elusive.

In the present study, we discovered a novel circRNA circZFR that was dramatically increased in BC tissues compared with adjacent normal tissues. High expression of circZFR was correlated with high pathological T stage, high grade, lymphatic metastasis, recurrence and poor prognosis, suggesting that circZFR acted as prognostic biomarker in BC. In addition, we first demonstrated circZFR served as an oncogene in BC through MTS assay, soft agar assay, cell cycle and cell apoptosis detection and transwell assay. Knockdown of circZFR could significantly diminish biological behavior of BC cells, implying that circZFR functioned as one oncogene and stimulated the development of BC.

Studies have manifested that circRNA functions as crucial gene regulators by their post-transcriptional modification such as binding miRNA, assembling RNA-binding proteins and modulating transcription factors [18]. CircRNA contains miRNA-binding site and usually exerts as a miRNA sponge to negatively regulate expression of target mRNAs [19]. For instance, circular RNA cTFRC acts as the sponge of miRNA-107 to promote bladder carcinoma progression [20]. Circular RNA circMTO1 suppresses BC metastasis by sponging miR-221 and inhibiting epithelial-to-mesenchymal transition [21]. Invasion-related circular RNA circFNDC3B inhibits BC progression through the miR-1178-3p/G3BP2/SRC/FAK axis [22]. In our study, bioinformatic analysis, biotin-labeled probe pull-down assay and dual-luciferase reporter assay confirmed that circZFR directly binds to miR-377. Additionally, we observed a negative relationship between circZFR and miR-377 in BC tissues. And miR-377 was significantly low-expressed in BC cells. Previous studies have demonstrated that miR-377 served as tumor suppressor to regulate the progression of lung cancer, cervical cancer, glioma, gastric cancer, esophageal cancer, hepatocellular carcinoma cells and clear cell renal cell carcinoma by targeting CD133, VEGF, Bcl-xL and ETS-1 [23–26]. Collectively, these data elucidated that circZFR exerted its oncogenic role by sponging miR-377.

Next, bioinformatics prediction and luciferase reporter assay revealed that miR-377 bound to ZEB2 and negatively regulated its expression. Previous evidences suggest that ZEB2 mediates oncogenic roles in BC. Wang et al. found that miR-454-3p and miR-374b-5p suppress migration and invasion of BC cells through targeting ZEB2 [27]. Liu et al. reported that down-regulation of long non-coding RNA TUG1 inhibits proliferation and induces apoptosis through the TUG1/miR-142/ZEB2 axis in BC cells [28]. In our study, we found that miR-377 targeted ZEB2 and suppressed its expression, whereas circZFR sponged miR-377 to promote ZEB2 expression in BC cells. Furthermore, through rescue assays, we confirmed that circZFR exerts its oncogenic role in a ZEB2-dependent manner.

In summary, our data provide comprehensive evidences that circZFR is a prognostic biomarker in BC. Furthermore, we also demonstrated that knockdown of circZFR could effectively prohibit cell proliferation and migration by targeting the miR-377/ZEB2 axis, suggesting a potential therapeutic target for circZFR in BC treatment.

## Supplementary Material

Supplementary Figure S1

## Abbreviations

BC: bladder cancer
CircRNA: circular RNA
OS: overall survival
PFS: progression-free survival
qRT-PCR: quantitative real-time polymerase chain reaction
