# Supplementary material for: CircZFR serves as a prognostic marker to promote bladder cancer progression by regulating miR-377/ZEB2 signaling
Source: Biosci Rep. 2019 Dec 4;39(12):BSR20192779. doi: 10.1042/BSR20192779 (PMC6893170; doi:10.1042/BSR20192779)
Supplement: Supplementary Figure S1 [file BSR-2019-2779_supp.pdf]

Figure S1

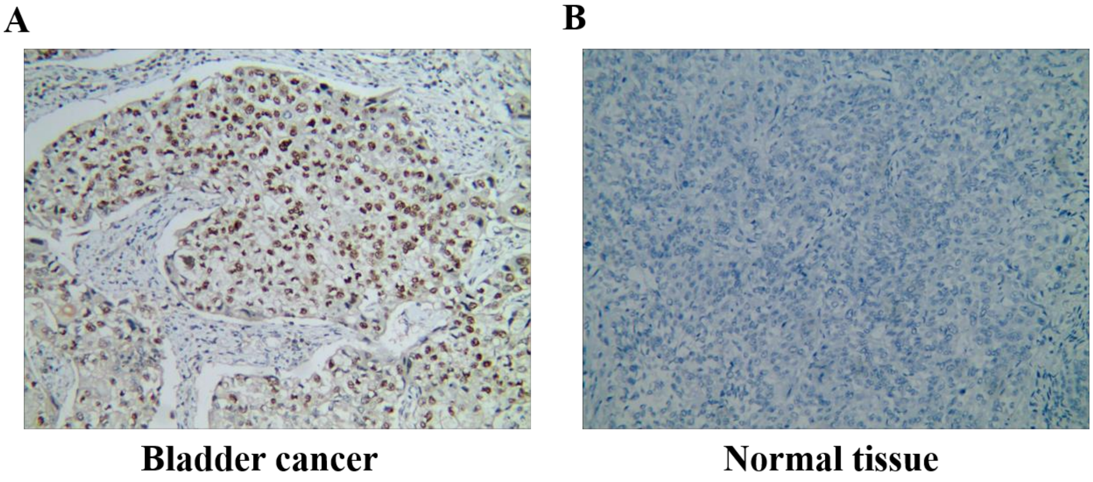

Figure S1 Immunohistochemistry staining was performed to detect the expression of ZEB2 in the bladder cancer and normal tissues.

The representative analytic results of ZEB2 immunohistochemistry staining of formalin-fixed, paraffin-embedded human bladder cancer tissue(A) and their paired adjacent noncancerous tissues from the same patient (B). (DAB stainning, original magnification × 100).
